# Touchscreen Response Precision Is Sensitive to the Explore/Exploit Trade-off

**DOI:** 10.1523/ENEURO.0538-24.2025

**Published:** 2025-05-06

**Authors:** Dana Mueller, Erin Giglio, Cathy S. Chen, Aspen Holm, R. Becket Ebitz, Nicola M. Grissom

**Affiliations:** ^1^Department of Psychology, University of Minnesota, Minneapolis, Minnesota 55455; ^2^Department of Neuroscience, University of Montreal, Montreal, Quebec H3T 1J4, Canada

**Keywords:** bandit, hidden Markov model (HMM), reinforcement learning, sex differences, touchscreen

## Abstract

The explore/exploit trade-off is a fundamental property of choice selection during reward-guided decision making, where the “same” choice can reflect either of these internal cognitive states. An unanswered question is whether the execution of a decision provides an underexplored measure of internal cognitive states. Touchscreens are increasingly used across species for cognitive testing and afford the ability to measure the precise location of choice touch responses. We examined how male and female mice in a restless bandit decision making task interacted with a touchscreen to determine if the explore/exploit trade-off, prior reward, and/or sex differences change the variability in the kinetics of touchscreen choices. During exploit states, successive touch responses are closer together than those made in an explore state, suggesting exploit states reflect periods of increased motor stereotypy. Although exploit decisions might be expected to be rewarded more frequently than explore decisions, we find that immediate past reward reduces choice variability independently of explore/exploit state. Male mice are more variable in their interactions with the touchscreen than females, even in low-variability trials such as exploit or following reward. These results suggest that as exploit behavior emerges in reward-guided decision making, all mice become less variable and more automated in both their choice and the actions taken to make that choice, but this occurs on a background of increased male variability. These data uncover the hidden potential for touchscreen decision making tasks to uncover the latent neural states that unite cognition and movement.

## Significance Statement

A given decision can be made for multiple reasons. While repetitions of a decision—such as right or left in a two choice task—may look similar to an outside observer, they may be generated by distinct internal cognitive states, such as the explore/exploit trade-off. Individuals may make a given decision to either explore its outcome or exploit its learned value. Here we employ the unique advantages of touchscreens to show that the explore/exploit trade-off changes the execution of the “same” decision and highlight persistent sex differences in motor variability. Touchscreens are increasingly ubiquitous in animal research and in human lives, and we highlight a novel measure of hidden cognitive states that is available via these devices.

## Introduction

Numerous tasks in neuroscience research ask animals or humans to repeatedly choose between two or more options based on differing sizes or probabilities of reward, to measure the neural processes of decision making. These sequential reward-guided decision making tasks are well known to engage explore/exploit trade-offs (Stephens, 2008; Addicott et al., 2017; Ebitz et al., 2018, 2019; Chen et al., 2021b, 2024; Wyatt et al., 2023). Across species, exploration represents periods of variable choice selection and heightened learning about the environment, while exploit behaviors show consistent, repeated choice selection that is less sensitive to trial-to-trial feedback (Daw et al., 2005; Frank and Fossella, 2011; Badre et al., 2012; Cavanagh et al., 2012; Trudel et al., 2021; Ting et al., 2023). The explore/exploit trade-off has been shown to differ across individual animals as a function of sex (Chen et al., 2021b; Glewwe et al., 2025) and in humans as a function of multiple neuropsychiatric diagnoses (Kaske et al., 2023; Speers and Bilkey, 2023; Knep et al., 2024; Lloyd et al., 2024; Yan et al., 2025), highlighting the impact of studying these latent cognitive states in preclinical testing.

Research on the explore/exploit trade-off reveals that the “same” decision or action in different trials, for example, choosing a left option over the right option in a two choice task, may be driven by differing cognitive strategies. Indeed, neural measures reveal that superficially similar choice behaviors can be driven by highly distinct neural states, including differences when animals are engaged in repeating choices (exploit) versus in sampling (explore; Ebitz et al., 2018, 2019, 2020; Tervo et al., 2021; Bolkan et al., 2022; Wang et al., 2023; Wyatt et al., 2023). Although explore and exploit strategies (and other latent cognitive states) are defined at the broadest level by the choice sequences in a decision making task and computational parameters derived from these choice sequences, these neural findings suggest that explore/exploit balance may also be reflected and measurable, in the fine-grained execution of a task, but this is largely unknown.

In contrast to typical rodent lever-press or nosepoke operant designs, touchscreen operant chambers offer a powerful and novel approach to measuring the fine-grained execution of a response by logging the precise coordinates and timing of each contact with the screen. Screens by default offer no immediate, tactile feedback about a choice, meaning that choices that are more or less variable in their location will be so because of internal states of the animal. Classic research with pigeons and other birds demonstrate their awareness of spatial location of touches on touchscreens and that they make minute adjustments of touches as a task evolves, suggesting that the same might be evident for rodents (Skinner, 1960; Goodale, 1983; Jager and Zeigler, 1991; Spetch et al., 1992; Capshew, 1993; Peterson, 2004). Rodent touchscreen tasks have increased in prevalence because they offer flexible and translational methods to assess cognition. These touchscreen approaches may also offer an underrecognized opportunity to assess latent or internal cognitive states such as the explore/exploit trade-off via measurements of the coordinates and timing of touch actions on the screen.

**Figure 1.**
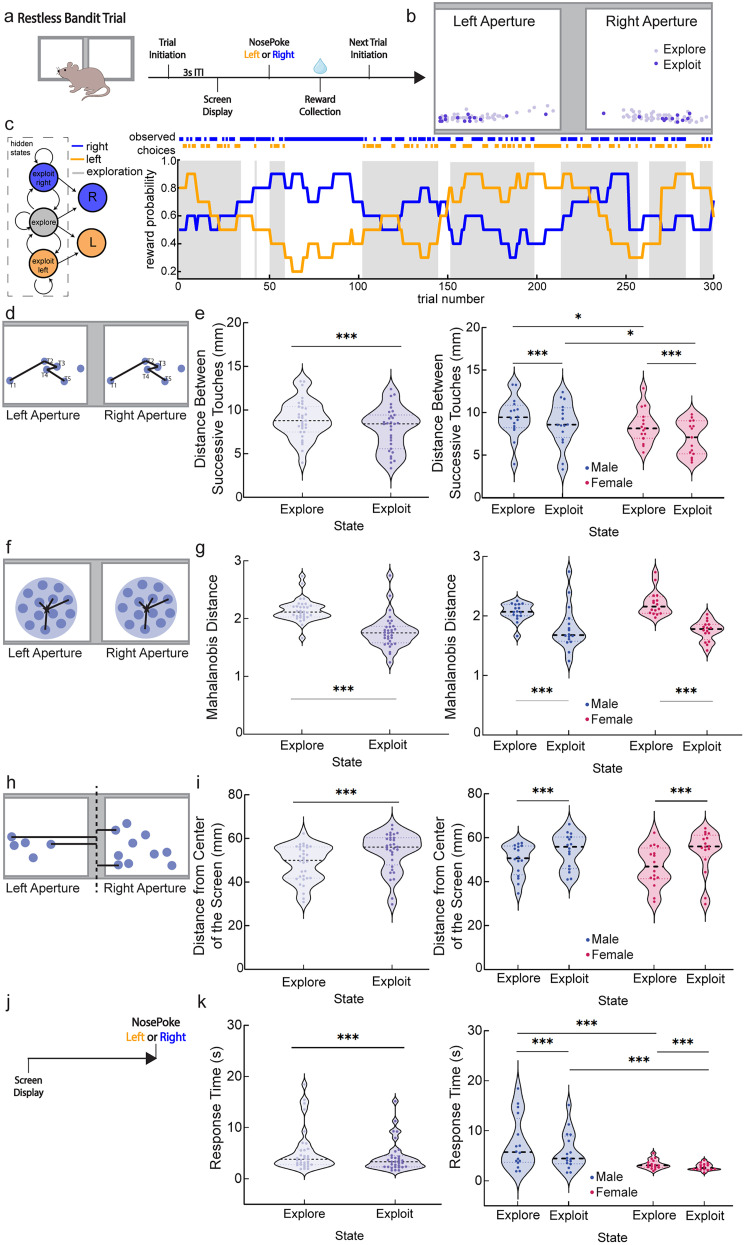
Exploit states and female sex reduce action variability during decision making. ***a***, schematic depicting the timeline of a single trial. White squares indicate left/right spatial choice. ***b***, An example of touchscreen responses from one animal and one session, where light purple indicates explore touches and dark purple indicates exploit touches. ***c***, Schematic depicting the HMM and labeling explore trials along an example two-arm spatial restless bandit probability walk. Orange traces indicate the probability and choices of left side touches. Blue traces indicate the probability and choices of right side touches. Gray-shaded regions indicate HMM-labeled explore trials. ***d***, Schematic of Euclidean distance where the distance is calculated between Touch 1 and Touch 2, Touch 2 and Touch 3, Touch 3 and Touch 4, and so on. Shown here are possible left/right touches in blue and the distance relationship from one to another represented by black lines. ***e***, Average Euclidean distance split by state (left) and sex (right). Exploit touches and females had significantly reduced Euclidean distance. Light purple indicates distance between explore touches, and dark purple indicates distance between exploit touches. Red indicates female, and blue indicates male mice. In violin graphs, individual data points are data from one mouse averaged across all sessions. ***f***, Schematic of Mahalanobis distance where the individual data points are measured from the overall centroid of the dataset. Shown here are possible left/right Mahalanobis clusters (light blue circles) and centroids (stars) and the Mahalanobis distance relationship from each touch (darker blue circles) in a cluster to the centroid represented by black lines. ***g***, Average Mahalanobis distance split by state (left) and sex (right). Exploit touches had significantly reduced Mahalanobis distance. Light purple indicates Mahalanobis distance between explore touches, and dark purple indicates Mahalanobis distance between exploit touches. Red indicates female, and blue indicates male mice. ***h***, Schematic of distance from the center of the screen where touch distance from both left and right choice apertures is measured from the midpoint of the operant screen. Shown here are possible left/right touches in blue and the distance of each from the center of the touchscreen indicated by black lines. ***i***, Average distance from the center of the screen split by state (left) and sex (right). Explore touches were significantly closer to the center of the screen. Light purple indicates distance from the center of the screen for explore touches, and dark purple indicates distance from the center of the screen for exploit touches. Red indicates female, and blue indicates male mice. ***j***, Schematic of response time calculation which is based on the difference between screen display and choice time (nosepoke). ***k***, Average choice response time split by state (left) and sex (right). Exploit touches and female sex significantly reduced latency to respond. Light purple indicates response time for explore touches, and dark purple indicates response time for exploit touches. Red indicates female and blue indicates male mice. For simplicity of visualization, all plots are averages across trials and sessions, so that each individual data point plotted represents the overall average for a mouse. Significance throughout this paper is represented in the following way: *p < 0.05 and *p > 0.01; **p < 0.01 and **p ≥ 0.001; ***p < 0.001. Violin graphs depict median and quartiles of the dataset.

We have previously used a touchscreen bandit decision making task to reveal robust sex differences in the explore/exploit trade-off in mice (Chen et al., 2021b). Here, we make use of previously unanalyzed touch location data from this experiment to uncover links between touchscreen interactions and the explore/exploit trade-off, prior reward, and sex differences. This task used a touchscreen configuration where a touch action anywhere within either of two large apertures would be recorded as a response. In this spatial bandit task, each of the two response areas (“arms”) was visually identical, and a left or right side choice was probabilistically rewarded (“spatial bandit”). The probability of reward on each of the two arms drifted slowly and independently of each other (“restless”) throughout each session—thus, a two-arm spatial restless bandit task. In this task, mice alternate between explore and exploit throughout the session (Ebitz et al., 2018; Chen et al., 2021b), as shifts in reward probability push the need to re-survey the choices or commit to one choice or the other when evidence suggests it is highly rewarding. We asked if explore/exploit balance in this task governed how variable or similar individual choice touches were from one trial to the next, given the wide possible space in which mice could indicate their choices (Fig. 1). We found that actions occurred much more similarly when made during exploit states compared with explore states, occurring closer together overall and across individual exploit states. Touch actions were also more similar following reward, an effect which was independent of explore/exploit balance, suggesting parallel mechanisms by which explore/exploit state and prior outcomes influence action execution. Furthermore, previous work from the lab has shown that male and female mice vary in their explore/exploit balance, such that males explore significantly more than females (Chen et al., 2021b). Here we find that touch actions are more variable in males overall compared with females, independent of both the impact of explore/exploit state and of reward experience, suggesting individual differences are also a key regulator of action execution over and above other cognitive influences. Overall, this novel analysis capitalizes on the hidden potential for touchscreens to measure not only choice behaviors but the motor actions that generate them, informing the neural states that unite movement and cognition.

## Materials and Methods

### Subjects

Behavioral data from these mice running this task were previously published by the lab (Chen et al., 2021b). Animals were 32 129/B6J F1 mice (16 males and 16 females) from The Jackson Laboratory. Colony rooms were temperature controlled (20.5°C; 69°F) and on a light/dark cycle of 12 h with the lights off at 9 A.M. Mice were housed in groups of four with water *ad libitum*. Mice were food restricted to no lower than 85% of their free-feeding body weight. All animals were cared for according to the guidelines of the National Institution of Health and University of Minnesota IACUC approval.

### Apparatus

Behavioral training and testing were carried out in the same touchscreen chambers for all mice throughout the present study (Lafayette Instrument Company). The sound-reducing chamber includes two black acrylic plastic walls with a touchscreen making up the third wall. The touchscreen was positioned directly opposite the reward port. Each chamber contained an automated food dispenser where 50% water-diluted vanilla Ensure was delivered. An opaque mask covered the screen with two response apertures for the training and behavioral task (Fig. 1*a*). All touchscreen choices were collected by the Lafayette ABET software.

### Behavioral training program

#### Chamber acclimation and schedules

Prior to the first day in the operant chamber, training mice were pre-exposed to vanilla Ensure in a bottle overnight allowing them familiarity with the reward prior to receiving the reward for the first time during a training schedule. The two-arm restless bandit task was preceded by a multicomponent training schedule. Mice completed the following training schedules: Day 0, initial touch, must touch, pairwise must initiate, pairwise punish incorrect, 100-0 deterministic learning training, 90-10 probabilistic learning training, 80-20 probabilistic learning training, and 70-30 probabilistic learning training. Day 0 is a single habituation day in the operant chamber where free reward (50 µl; vanilla Ensure) is given at the very beginning of a 30 min exposure to the operant chamber.

#### Initial touch training

This training trains mice to use the touchscreen. A free reward (7 µl) is given every 30 s; however, if a mouse touches the random image on the screen, it will get an additional immediate reward dispensed which is three times the amount of the free reward (21 µl). This training schedule lasts 30 min.

#### Must touch training

This training requires mice to use the touchscreen to gain rewards. There is no longer a free reward every 30 s, but rather if a mouse touches the random image on the screen, immediate reward is dispensed (7 µl). This training schedule lasts 30 min.

#### Pairwise must initiate training

This training trains mice to initiate a trial by entering the reward port. At this stage of training, mice have learned to touch the screen after it has been lit to gain a reward (7 µl). The reward is followed by a 3 s intertrial interval (ITI), and then a light cue at the reward port is turned on to signal the mouse to enter the reward port to initiate a new trial. This training schedule lasts 30 min.

#### Pairwise punish incorrect training

This training trains the precision of touchscreen response to the lit-up image only (not any part of the screen). If a mouse wrongly touches an unlit portion of the screen (e.g., left side instead of right side), the operant chamber house light will blink for 2 s, and there will be a 10 s timeout as punishment. If the mouse correctly touches the lit portion of the screen, they are rewarded (7 µl), followed by a 3 s ITI, and the mouse must enter the reward port to initiate a new trial. This training schedule lasts 60 min or until 200 trials.

#### 100-0 deterministic learning training

This deterministic training schedule is the first value-based decision making training that requires a mouse to choose between two images and learn about the correct image from feedback. One image is rewarded 100% of the time, and the other image is rewarded 0% of the time with no punishment timeout. The rewarded image switches between the left and right side but is always rewarded regardless of spatial location. This training schedule lasts 120 min or until 250 trials.

#### Probabilistic learning training

This training consists of a series of probabilistic reversal learning schedules. A 90-10 spatial training requires a mouse to choose between the left and right side (identical visual cue), where one side is rewarded 90% of the time and the other side is rewarded 10% of the time. The 80-20 and 70-30 spatial trainings are the same with 80 versus 20% rewarded options and 70 versus 30% rewarded options. The reward probability associated with the left and right side will reverse based on choice matching probability of reward, e.g., 90-10 reversal occurs after the high-value choice is chosen 9 out of the last 10 trials. Mice experienced each probabilistic schedule for one session in the following order: 90-10, 80-20, and 70-30 prior to two-arm spatial restless bandit testing. The purpose of this training is to adapt mice to a stochastic and changing environment, prior to the restless bandit task.

### Restless bandit behavioral paradigm

In this version of the bandit task (Chen et al., 2021b), mice must decide between two choices (left or right) on a touchscreen which present as illuminated white squares and are associated with some probability of reward that changes independently and randomly over time (Fig. 1*a*). A nosepoke to the touchscreen is required to register a choice response. On every trial, there is a 10% chance of the reward probability associated with each arm increasing or decreasing by 10%. The reward contingency is always stochastic, which means the reward probability cannot go down to 0% or up to 100% and was limited to a minimum of 20% and a max of 90% (Fig. 1*c*). Figure 1*c* shows an example of a probability walk. Each day of a two-arm spatial restless bandit consisted of a new walk of independent and randomly changing probabilities to require new learning of contingencies daily. Rewarded responses received vanilla Ensure reward at the reward port at the rear of the chamber (∼7 µl). Mice completed either 300 trials or spent a maximum amount of 2 h in the operant chamber each day. All choice sequences (right or left touch, *x*, *y* coordinates of each touch, response times) were collected by the Lafayette ABET software.

Computational models were previously fit to the data from these animals, including a hidden Markov model (HMM) and a reinforcement learning choice kernel (RLCK) model (Chen et al., 2021b). The HMM was used to determine when animals were exploring or exploiting their options in the two-arm spatial restless bandit task, where P(exploration) is the probability of mouse exploration between choices. Figure 1*c* (left) uses arrows to represent the possible state transitions determined by the HMM, where a decision state can remain the same or a transition from explore to exploit or exploit to explore can occur. Figure 1*c* also shows that a transition from exploiting one side to the other cannot be made without entering a period of exploration first. Figure 1*c* (right) is an example probability walk with HMM state assignment overlayed. Orange tick marks at the top of the figure indicate a choice made on the left side by the mouse for that specific trial. Blue tick marks at the top of the figure indicate a choice made on the right side by the mouse for that specific trial. The orange and blue lines tracing across 300 trials indicate the reward probability for the left and right side, respectively, across each trial of the session. Gray-shaded regions indicate HMM-labeled explore trials (Fig. 1*c*). The previous manuscript compared several different RL models and identified the strongest fit to animal behavior from an RLCK model, which captures both value-based and value-independent decisions using the following four parameters: learning rate, decision noise, choice bias, and choice stickiness. Here we use this RLCK model's alpha parameter compared with distance between successive touches to assess how the learning rate impacts micro adjustments to spatial touch locations across sex. For validation of both models, please see Chen et al. (2021b).

### Coordinate analysis

The Bussey–Saksida touchscreen apparatus (Lafayette Instrument Company) is sensitive to continuous and rapidly repeated touches in the same location and across the entirety of the screen (Heath et al., 2015). Each touchscreen represents the *x* and *y* coordinates of each response an animal makes on the screen from IR beam technology where IR emitters are positioned along two sides of the screen (i.e., top and right sides) and IR receivers are positioned along the other two sides of the screen (i.e., bottom and left sides). In this configuration, IR beams are ideally suited to determine the shadow of the touch to triangulate the location of choice response. IR beam configuration results in a touch resolution that matches the monitor resolution of 800 × 600 pixels. Figure 1*b* visualizes these data, representing the choices an example mouse selects between two options on the touchscreen over 300 trials, with explore responses in the lighter purple and exploit responses in the darker purple. Figure 1*b* provides an example of nosepoke responses for one mouse across a single session and the change in touch pattern between explore/exploit touches as identified by our HMM. Left and right touchscreen choice apertures are 240 × 240 pixels each, never change position or size, and *x* and *y* coordinates are separately generated for each touch aperture. Throughout all analyses, we have transformed pixels into millimeters. One pixel is 0.29 mm. Unless mentioned otherwise, for all data, a generalized linear mixed model (GLMM) stepwise model selection analysis was used to determine the optimal model with the lowest AIC value, and *p* values are shared from those most optimal models.

### Distance from the center of the screen

The spatial split in exploration and exploitation visualized by these plots (Fig. 1*b*) suggested that explore trials were closer to the center of the touchscreen than exploit trials were, prompting us to quantify the distances (Fig. 1*i*). With the center of the screen being 400 out of 800 total pixels (width of the screen), the difference between the *x* pixel coordinate of the *x* and *y* location of each touch response and 400 pixels was calculated and converted into millimeters. An absolute value is applied so that the distance away from the center of the screen is always a positive value to reflect distance. This calculation was done across all touches in every session. Trials were split by explore and exploit, and all data were averaged across all eight two-arm spatial restless bandit sessions for graphing purposes.

Example(x,y)is(34,208),

Distancefromthecenterofthescreen=|400−x|,

Distancefromthecenterofthescreen=|400−34|=366pixels.

### Euclidean analysis

The first method we used to quantify the distance between nosepoke touches was a Euclidean analysis (Walther et al., 2016; Ebitz and Hayden, 2021) in which we used the Pythagorean theorem to calculate the hypotenuse between two points with (*x*, *y*) coordinates that were successive, from the same choice aperture (left/right), and within the same HMM decision state (explore/exploit; Fig. 1*d*). In Python, this calculation was done using numpy.hypot(). A drawback of this analysis is the amount of data points that get excluded given that the included data points must be consecutively from the same choice aperture side and within the same state. In total, 37% of trial choices are omitted because of these transitions. A 35.3% of excluded trial choices displays side (left/right aperture) transitions, while 8.2% of excluded trial choices displays state (explore/exploit) transitions, with a portion of excluded trial choices including both state and side transitions. Distances were split by explore and exploit, and all data were averaged across all eight two-arm spatial restless bandit sessions for graphing purposes. In the example below, “*T*” represents touch (nosepoke):

$$\mathrm{Example}\;T_{1}\;\mathrm{is}\;(x_{1},y_{1})\;\mathrm{and}\;T_{2}\;\mathrm{is}\;(x_{2},y_{2}),$$

$$\mathrm{Distance}\;\mathrm{between}\;\mathrm{successive}\;\mathrm{touches}\;(\mathrm{hypotenuse})=\surd ({\left(x_{2}-x_{1}\right)}^{2}+{\left(y_{2}-y_{1}\right)}^{2}).$$

### Mahalanobis analysis

The second method we used to quantify touch patterns was a Mahalanobis analysis (Walther et al., 2016; Ebitz and Hayden, 2021) where, unlike the Euclidean analysis, we did not have to exclude any touch data points. With this analysis, we were able to calculate separate centroids based on the data clusters for both the left side touches and right side touches and calculate the distance of each touch coordinate from each overall centroid (Fig. 1*f*). The centroid is the central point in the data field that can be considered the overall mean for multivariate data given that this is the point where all means from all variables intersect. The further away a data point (touch) is from the centroid, the larger the Mahalanobis distance value. Distances were split by explore and exploit, and all data were averaged across all eight two-arm spatial restless bandit sessions for graphing purposes. In the formula below, *X*_A_ and *X*_B_ represent a pair of objects, which are the *x* and *y* coordinates; *C* is the sample covariance matrix, calculated using numpy.cov() in python; and *T* is the transposition of the matrix over its diagonal, calculated using numpy.linalg.inv() in Python:

$$\mathrm{Mahalanobis}\;\mathrm{distance}\;=\;[{\left(X_{B}-X_{A}\right)}^{T}\;*\;C^{-1}\;*\;(X_{B}-X_{A}){]}^{0.5}.$$

### Latency to respond

To determine whether latency to respond in the two-arm spatial restless bandit task differs by state and sex, we calculated the response time in seconds. The response time was calculated as the time elapsed between the screen display onset and the time when the nosepoke to the left or right choice aperture was completed (Fig. 1*J*).

### Reward

To determine whether being rewarded in the two-arm spatial restless bandit task impacts touch location, we compared trial outcome (rewarded or nonrewarded) from the previous trial (*T*_−1_) to the change in touch location on the current trial (*T*_0_). This was done using both Euclidean and Mahalanobis analyses.

### Distance between successive bouts

To understand how touches were organized within and across periods of exploration or exploitation as defined by HMM, we divided the data into “bouts.” Rather than looking at our nosepoke data clusters throughout an entire session, a “bout” is described as a period of touches within one HMM-defined behavioral state on one particular choice aperture. Thus, explore states may contain separate bouts on the left or right side, but these are analyzed separately. State transition trials from either explore to exploit or exploit to explore trigger a new “bout.” By looking at individual state bouts of choice responding, we can investigate whether explore or exploit centroids on a given response area are shifting more throughout a session. This analysis combines both Euclidean and Mahalanobis methods previously described. Mahalanobis analysis is used to determine the centroid of each individual “bout.” From here, the distance between successive centroids is calculated using the Euclidean analysis, which employs the Pythagorean theorem (Fig. 3*f*). Distances were split by explore and exploit, and all data were averaged across all eight two-arm spatial restless bandit sessions for graphing purposes. In the example below, “*C*” represents centroid:

$$\mathrm{Example}\;C_{1}\;\mathrm{is}\;(x_{1},y_{1})\;\mathrm{and}\;C_{2}\;\mathrm{is}\;(x_{2},y_{2}),$$

$$\mathrm{Distance}\;\mathrm{between}\;\mathrm{successive}\;\mathrm{touches}\;(\mathrm{hypotenuse})=\surd ({\left(x_{2}-x_{1}\right)}^{2}+{\left(y_{2}-y_{1}\right)}^{2}).$$

### Contour plots and area calculations

In order to calculate the amount of space occupied by each bout, we calculated the area and perimeter of the bouts. In Python, 2D contour plots from Plotly Graphing Libraries were fit over our nosepoke touch locations to visualize the density and range of choice responding. Bins edges were designated by numpy.histogram and filtered at every-other bin, so they were twice as big as the standard output. The color bar was fixed from 0 to 1 across all generated plots to ensure consistency of calculations (Fig. 3*c*). Contour fill was removed, leaving just the outlines at a thickness of “3,” so the trace would be better recognized by OpenCV.

Once a contour plot was generated for each bout, Open Source Computer Vision (OpenCV) was used to capture the contours along continuous boundaries and calculate area (cv.contourArea) and perimeter (cv.arcLength) for each bin. While tracing the contours, cv.threshold was set to cv.THRESH_BINARY, and cv.findContours was set to cv.CHAIN_APPROX_SIMPLE. Contour Approximation was used when it was necessary to approximate the area between two separate contour groups. We focused on the dimensions of the outermost bin as the best representation for the spread of data throughout a bout (Fig. 3*c*). The outermost bin was filtered using the structure hierarchy or rather the nested orientation of the contours labeled numerically with “parent” and “child” identifications. Areas and perimeters of bouts were split by explore and exploit, and all data were averaged across all eight two-arm spatial restless bandit sessions for graphing purposes. Finally, the area and perimeter were calculated for the correctly identified contour bin. OpenCV was run through the University Supercomputing Institute.

### Data analysis

Data were analyzed with custom Python and GraphPad Prism 10 scripts. GLMM (package pymer4 in Python) were used to determine the state, sex, and reward differences over time, unless otherwise specified (Jolly, 2018). *P* values were compared against the standard *α* = 0.05 threshold. Significance throughout this paper is represented in the following way: *p < 0.05 and *p > 0.01; **p < 0.01 and **p ≥ 0.001; ***p < 0.001. The sample size is *n* = 16 for both males and females for all statistical tests. No animal was excluded from the experiment. All statistical tests used and statistical details were reported in the results. For simplicity of visualization, all plots are averages across trials and sessions, so that each individual data point plotted represents the overall average for a single mouse. Violin graphs depict median and quartiles of the dataset.

Winning models were selected using a stepwise GLMM approach starting by including sex and state as categorical fixed variables and individual mouse identity as a categorical random variable—as well as all pairwise interactions between the three. During the model selection process, each child model was created by dropping one variable or interaction from the parent model and choosing the model with the lowest AIC until no drops in AIC were observed without completely dropping significant main effects. In Table 1, we report all effects of the model with the lowest AIC for each analysis.

**Table 1.** Generalized linear mixed models Equations 1–12

Variable	Coded as	GLMM series	Dependent variable	Figure	Beta Coeff.	*P* value
dist_mm∼sex×state+(state|mouseID),						
(state|mouseID)	Random	Euclidean	dist_mm	1*e*	10.42632	3.52 × 10^−11^
Sex	Categorical	Euclidean	dist_mm	1*e*	2.668432	0.012418
State	Categorical	Euclidean	dist_mm	1*e*	4.479342	0.000118
Sex × state	Interaction	Euclidean	dist_mm	1*e*	−1.63685	0.112654
mahalanobis∼state+(state|mouseID),						
(state|mouseID)	Random	Mahalanobis	mahalanobis	1*g*	42.47032	1.27 × 10^−29^
State	Categorical	Mahalanobis	mahalanobis	1*g*	6.132201	2.03 × 10^−6^
center_dist_mm∼sextimesstate+(state|mouseID),						
(state|mouseID)	Random	Distance From Center	cent_dist_mm	1*i*	24.75777	1.70 × 10^−21^
Sex	Categorical	Distance From Center	cent_dist_mm	1*i*	0.299544	0.766587
State	Categorical	Distance From Center	cent_dist_mm	1*i*	−4.72546	5.15 × 10^−5^
Sex × state	Interaction	Distance From Center	cent_dist_mm	1*i*	0.767197	0.448969
dist_mm∼reward+(reward|mouseID)+(state|mouseID)+sextimesstate,						
(state|mouseID)	Random	Euclidean	dist_mm	2*a,b*	11.7435	1.04 × 10^−12^
Sex	Categorical	Euclidean	dist_mm	2*b*	2.779354	0.009519
State	Categorical	Euclidean	dist_mm	2*a*	−0.30298	0.764276
Reward	Categorical	Euclidean	dist_mm	2*a,b*	−6.96119	9.88 × 10^−8^
Sex × state	Interaction	Euclidean	dist_mm	2*a,b*	−2.17184	0.038554
mahalanobis∼reward+state+(state|mouseID)+(reward|mouseID),						
(reward|mouseID)	Random	Mahalanobis	mahalanobis	2*c,d*	29.75193	3.78 × 10^−38^
State	Categorical	Mahalanobis	mahalanobis	2*c*	4.383663	0.000181
Reward	Categorical	Mahalanobis	mahalanobis	2*c,d*	−4.01022	0.000364
loc_dist2_lastLoc∼statetimessextimes(1|mouseID),						
(1|mouseID)	Random	Local centroid distance	loc_dist2_lastLoc	3*g*	10.73884	1.18 × 10^−11^
Sex	Categorical	Local centroid distance	loc_dist2_lastLoc	3*g*	−0.01149	0.99088
State	Categorical	Local centroid distance	loc_dist2_lastLoc	3*g*	8.964287	4.03 × 10^−19^
State × sex	Interaction	Local centroid distance	loc_dist2_lastLoc	3*g*	2.078314	0.03772
contour_area∼statetimessex+(1|mouseID),						
(1|mouseID)	Random	Average Bout Area	contour_area	3*d*	5.058701	1.95 × 10^−5^
Sex	Categorical	Average Bout Area	contour_area	3*d*	4.731083	4.57 × 10^−5^
State	Categorical	Average Bout Area	contour_area	3*d*	−2.661	0.00781
State × sex	Interaction	Average Bout Area	contour_area	3*d*	−2.87523	0.004051
contour_perimeter∼statetimessex×+(1|mouseID),						
(1|mouseID)	Random	Average Bout Perimeter	contour_perimeter	3*e*	13.83109	1.05 × 10^−14^
Sex	Categorical	Average Bout Perimeter	contour_perimeter	3*e*	3.643466	0.000928
State	Categorical	Average Bout Perimeter	contour_perimeter	3*e*	−7.16055	8.93 × 10^−13^
State × sex	Interaction	Average Bout Perimeter	contour_perimeter	3*e*	−1.53537	0.124742
RT∼(state|mouseID)+state+sex,						
(state|mouseID)	Random	Response Latency	RT	1*k*	8.731039	1.23 × 10^−9^
Sex	Categorical	Response Latency	RT	1*k*	3.891523	0.000528
State	Categorical	Response Latency	RT	1*k*	4.54362	8.54 × 10^−5^
distance∼alpha+sex+(1|mouseID),						
(1|mouseID)	Random	Learning Rate	alpha	N/A	11.52081	1.76 × 10^−14^
Sex	Categorical	Learning Rate	alpha	N/A	2.512188	0.017736
Alpha	Continuous	Learning Rate	alpha	N/A	−2.01035	0.045508
num_explore∼sex+(1|mouseID),						
(1|mouseID)	Random	Bout number	num_explore	3*b*	12.12988	6.87 × 10^−13^
Sex	Categorical	Bout number	num_explore	3*b*	−2.1784	0.037647
num_exploit∼sex+(1|mouseID),						
(1|mouseID)	Random	Bout number	num_exploit	3*b*	10.75028	1.23 × 10^−11^
Sex	Categorical	Bout number	num_exploit	3*b*	−2.36733	0.024802

Table including equations, variables, figure references, beta coefficients, and *p* values for all included statistics.

**Figure 2.**
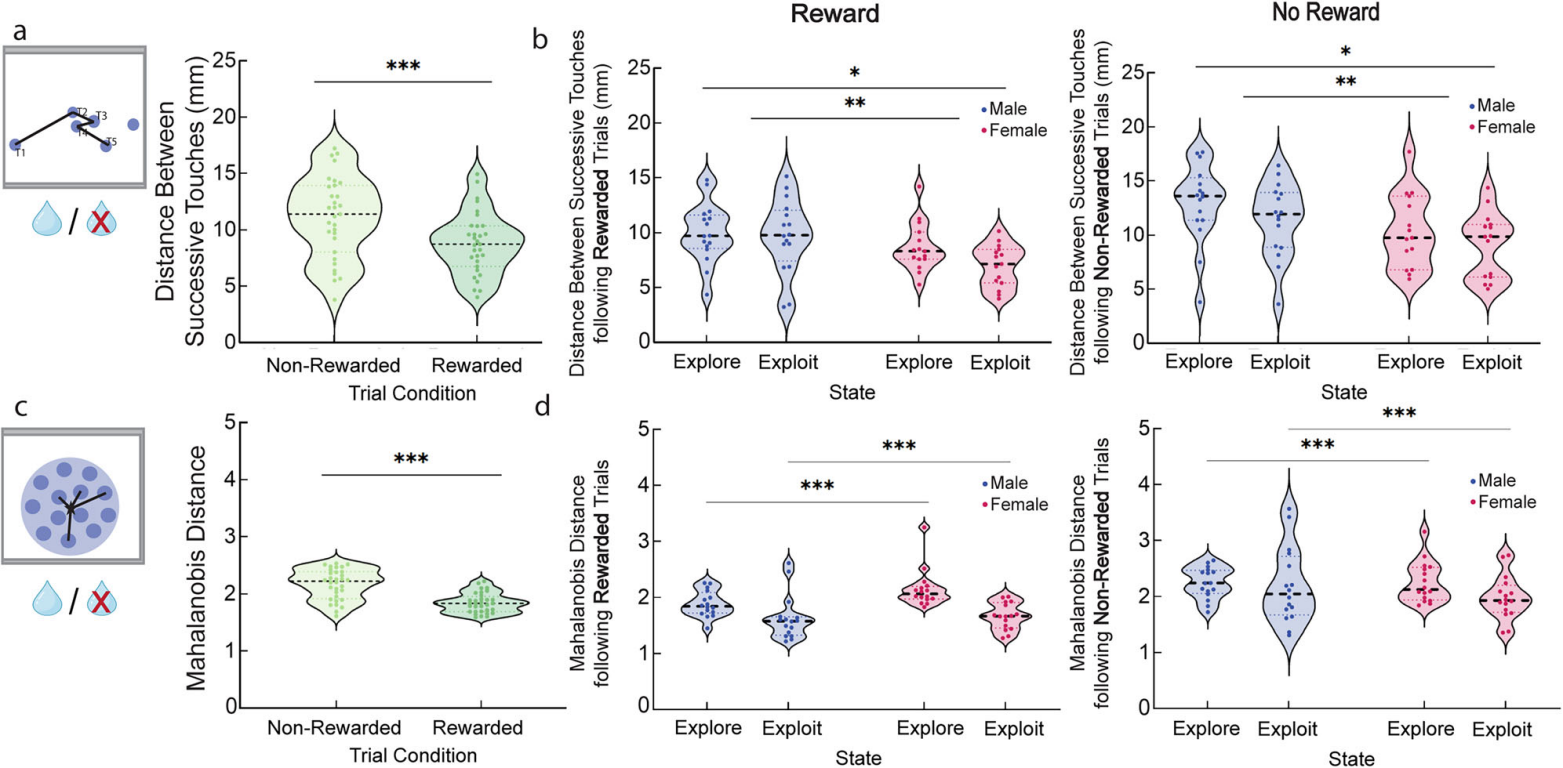
Previous reward reduces action variability independently from explore/exploit balance or female sex. ***a***, Average Euclidean distance comparing rewarded versus nonrewarded trials. Touches following rewarded trials had significantly reduced Euclidean distance. Light green indicates distance between nonrewarded touches, and dark green indicates distance between rewarded touches. In violin graphs, individual data points are data from one mouse averaged across all sessions. ***b***, Average Euclidean distance for rewarded (left) and nonrewarded (right) trials split by state and sex. Exploit touches and females had significantly reduced Euclidean distance. Red indicates female, and blue indicates male mice. ***c***, Average Mahalanobis distance comparing rewarded versus nonrewarded trials. Touches following rewarded trials had significantly reduced Mahalanobis distance. Light green indicates Mahalanobis distance between nonrewarded touches, and dark green indicates Mahalanobis distance between rewarded touches. ***d***, Average Mahalanobis distance for rewarded (left) and nonrewarded (right) trials split by state and sex. Exploit touches had significantly reduced Mahalanobis distance. Red indicates female, and blue indicates male mice. *p < 0.05 and *p > 0.01; **p < 0.01 and **p ≥ 0.001; ***p < 0.001. Violin graphs depict median and quartiles of the dataset.

When considering the impact of reward (relevant to Fig. 2), we used a similar stepwise GLMM method, except that the previous reward (i.e., whether the mouse had been rewarded on the trial before the focal trial in which a decision was made) was also included as a fixed categorical factor along with all potential pairwise interactions between the previous reward and sex, state, and individual mouse identity in the starting model. Thereafter, we removed parameters in a stepwise pattern in the same way as previously described.

### Code accessibility

Codes used can be found at https://doi.org/10.5061/dryad.31zcrjdxt, with full accessibility for all interested parties. Included in this repository are all raw ABET behavioral data, all processed data with HMM trial labels, and all reinforcement learning model (RLCK) output originally published in Chen et al. (2021b). Also included are all custom Python scripts necessary for repeating our novel touchscreen analyses including but not limited to Euclidean, Mahalanobis, distance from the center, and “bout” labeling calculations. Finally, we have included all the code necessary to generate statistical results. If this code is applied to new datasets, please cite this paper.

## Results

To understand how actions in a touchscreen decision making task are influenced by internal decision making states, we took advantage of a previously collected dataset examining sex differences in explore/exploit balance in mice in a touchscreen two-arm spatial restless bandit task. Decision making data from this novel bandit task were originally shared, and modeling results are described in Chen et al. (2021b). These data were collected from age-matched male and female wild-type mice (*n* = 32, 16 per sex, strain B6129SF1/J). Mice were trained in a two-arm spatial restless bandit task (Fig. 1*a*,*c*) in a trapezoidal-shaped touchscreen operant chamber. In this two-arm spatial restless bandit task, the probability of reward of each left and right choice changes independently and randomly of the other, with a 10% chance of probability change on each trial (Fig. 1*c*, example probability walk). The unpredictability of this task encourages mice to continually learn and survey their choices, exploring to find the best option and exploiting a good rewarding option across a 300 trial session. Explore and exploit trials were labeled using an HMM approach (Ebitz et al., 2018; Chen et al., 2021b) where each trial was defined as either an explore choice or an exploit choice on the left or the right (Fig. 1*c*). Mice explore between the two choices or exploit the high-value choice throughout each session in order to maximize reward. The HMM is structured such that a mouse cannot go directly from an exploit state for one choice aperture to an exploit state for the other without entering a state of exploration. Due to the randomly changing probabilities throughout the task, mice must continually learn across 300 trials rather than just at the beginning of the session, and thus all mice continually transition between explore and exploit states in each session. Each trial nosepoke response on the touchscreen can therefore be identified as an explore or exploit choice (Fig. 1*b*).

### Exploit states and female sex are associated with reduced action variability

Using previously assigned explore/exploit states for each trial, we examined the action associated with each choice, taking advantage of logging the coordinate locations of nosepokes in our touchscreen operant chambers. This allowed us to have a two-dimensional location for each decision a mouse made across the entire touchscreen space. We started with an Euclidean analysis to quantify the distance between successive touch responses where T1 (touch/nosepoke 1) was compared with T2 (touch/nosepoke 2), T2 was compared with T3, T3 was compared with T4, so long as all touches were from the same choice aperture and state (Fig. 1*d*; Walther et al., 2016; Ebitz and Hayden, 2021). One mouse was excluded from Euclidean analyses as they never had a sequence of choices on the same side in the same state consecutively. During exploit states, successive choices were closer in space on the touchscreen than during explore states (Fig. 1*e*, GLMM, main effect of state; *p* < 0.001; *β*_state_ = 4.479; see Eq. 1 in Table 1). However, sex also played a role—female mice had shorter distances between successive touches than male mice (Fig. 1*e*, GLMM, main effect of sex; *p* = 0.01; *β*_sex_ = 2.668; see Eq. 1 in Table 1). The model used included an interaction term between the state and sex, which was not significant (Fig. 1*e*, GLMM, interaction state/sex; *p* = 0.113; *β*_sex * state_ = −1.637; see Eq. 1 in Table 1). These data argue that exploit states and female sex are independently associated with more similar, repeatable actions across sequential decision making.

Although these data suggest that exploit choices are more stereotyped than exploration, Euclidean analysis can only compare distances between touches that are consecutively occurring on the same side and in the same explore/exploit state. An alternative approach for calculating distance that permits all touches to remain in analysis is the Mahalanobis distance, a method for finding the distance between a point and the center of a distribution (Fig. 1*f*; Walther et al., 2016; Ebitz and Hayden, 2021). With Mahalanobis distance, the entire cluster of data points was analyzed for each choice aperture, including both explore and exploit touches. We separated the population of touch responses into those happening in explore states and those in exploit states and calculated separate Mahalanobis distances for exploit and explore touches from centroids within each left/right choice aperture, combining the data from both apertures across all trials and sessions and getting an average distance for each animal. The Mahalanobis distance of an average exploit touch from the centroid of all exploit touches was smaller and less variable than the distance of an average explore touch from the explore centroid (Fig. 1*g*, GLMM, main effect of state; *p* < 0.001; *β*_state_ = 6.132; see Eq. 2 in Table 1). Unlike Euclidean analysis, we do not find significant sex differences in Mahalanobis distances (sex was dropped in the GLMM with the lowest AIC value). The difference between sex influences on Euclidean and Mahalanobis distances may reflect the trial-to-trial variability that Euclidean analysis captures versus the overall distribution captured by Mahalanobis analysis. However, both analyses reveal a main effect of explore/exploit state on touch variability—that exploit touches occur closer together in space with less variability than explore touches.

In maze tasks, as animals approach a choice point, they exhibit a behavior called vicarious trial and error in which they move their head while surveying options to guide flexible decision making, which is reduced as choices become repetitive (Tolman, 1939, 1948; Johnson and Redish, 2007; Redish, 2016; George et al., 2023). This raised the possibility that in a touchscreen environment, flexible decision making may be reflected in the approach to the screen, allowing them to survey choices from a central location while exploring versus approaching directly toward one option when exploiting. To determine whether our mice might be exhibiting physical signs of deliberation between the left and right choice apertures during the explore state, we calculated the distance from the midpoint of the entire touchscreen between the two response apertures (Fig. 1*h*). Explore touches happen significantly closer to the center of the screen and thus closer to the opposite response aperture than exploit touches (Fig. 1*i*, GLMM, main effect of state; *p* < 0.001; *β*_state_ = −4.725; see Eq. 3 in Table 1). This did not differ by sex (GLMM, no main effect of sex; *p* = 0.767; *β*_sex_ = 0.300; see Eq. 3 in Table 1). The model used included an interaction term between state and sex, which was not significant (Fig. 1*i*, GLMM, state/sex; *p* = 0.449; *β*_sex*state_ = 0.767; see Eq. 3 in Table 1). These results suggest that in an explore state, mice exhibit a vicarious trial-and-error–like behavior as they approach an area equidistant from both response apertures and deliberate between left and right choice. Conversely, in an exploitative state, mice make responses committed to one aperture at a farther distance from the center of the screen.

Animals could show reduced variability in their touch responses across explore/exploit state and sex for two reasons. One possibility is that animals are expending increased effort to improve their accuracy, in which case we might expect slowed responses when touches are closer together. Alternatively, increased similarity in touch locations could result from increased behavioral automaticity, which would be expected to be associated with increased speed for touches with increased accuracy in exploit states and in females compared with males. We find evidence to support the latter hypothesis. Response time (Fig. 1*j*) in exploit state was smaller and therefore quicker than response time in explore state (Fig. 1*k*, GLMM, main effect of state; *p* < 0.000; *β*_state_ = 4.544; see Eq. 9 in Table 1). Sex also played a role—female mice had quicker response times than male mice (Fig. 1*k*, GLMM, main effect of sex; *p* < 0.001; *β*_sex_ = 3.892; see Eq. 9 in Table 1). These results suggest that exploit choices represent a more automated, stereotyped behavioral response than the same choice made during exploration and suggest that these behaviors are more stereotyped overall in female mice compared with males.

### Previous reward is associated with reduced action variability separate from the effect of explore/exploit state

One potentially significant difference between explore and exploit states that might influence animal actions are differing reward rates across states. Exploit behavior is likely to result from prior success in obtaining reward, and thus exploit states might be expected to be associated with higher reward. Alternatively, reward may have a separate impact on action variability that is unrelated to explore/exploit state influences (Trommershäuser et al., 2003; Abe et al., 2011; Izawa and Shadmehr, 2011; Galea et al., 2015; Hasson et al., 2015; Nikooyan and Ahmed, 2015; Ramkumar et al., 2016; Therrien et al., 2016; Cashaback et al., 2017). To examine the impact of reward on touch location, we separated trials by outcome: rewarded/not rewarded. To determine the impact of being rewarded on a previous trial, we have taken the distance measurements between one trial back (*T*_−1_)—labeled as “rewarded” or “nonrewarded”—and the current trial (*T*_0_). Euclidean and Mahalanobis distances for touches on trials following rewarded choices was smaller and less variable than those following nonrewarded touches (Fig. 2*a*, GLMM, main effect of reward; *p* < 0.001’ *β*_reward_ = −6.961; see Eq. 4 in Table 1; Fig. 2*c*, GLMM, main effect of reward; *p* < 0.001; *β*_reward_ = −4.010; see Eq. 5 in Table 1). However, the effect of reward on action variability was independent of an effect of explore/exploit state on action variability, with both previous trial reward and explore/exploit state contributing main effects on the variability of choice responses (Fig. 2*b*, GLMM, main effect of reward; *p* < 0.001; *β*_reward_ = −6.961; see Eq. 4 in Table 1; Fig. 2*d*, GLMM, main effect of state; *p* < 0.001; *β*_state_ = −4.384; see Eq. 5 in Table 1). Euclidean effects were stronger in females (Fig. 2*b*, GLMM, main effect of sex; *p* = 0.01; *β*_sex_ = 2.779; see Eq. 4 in Table 1; and a sex by state interaction Fig. 2*b*, GLMM, sex/state interaction; *p* = 0.039; *β*_sex*state_ = −2.172; see Eq. 4 in Table 1). As expected from prior Mahalanobis analysis, there was no influence of sex on Mahalanobis distances. These results suggest that while reward is associated with increased precision/decreased variability in responding on the touchscreen, it is independent of the increased automaticity driven by exploit states and sex shown in Figure 1.

Our analysis suggests that rewards are an independent contributor to action variability from exploit states. This raises the question of whether sensitivity to reward parametrically influences action variability. To measure this, we took advantage of previously calculated reinforcement learning models from Chen et al. (2021b), focusing on the “value updating” or “learning rate” parameter alpha. We reasoned that because the Euclidean distance between touches is a measure of trial-to-trial action variability, this might relate to trial-to-trial value updating measured by alpha. Indeed, we previously found in the animals in the current dataset that the alpha parameter was significantly higher in females, suggesting greater trial-to-trial influences of outcome on a female mouse's next choice than on a male's. Therefore, we asked whether trial-to-trial action variability as measured by Euclidean distance between sequential touches on either aperture was correlated with trial-to-trial outcome sensitivity as measured by the alpha parameter for the best fit reinforcement learning model from Chen et al. (2021b). With sex, distance, and alpha parameters as fixed effects and individual mouse as a random effect, the GLMM revealed that a higher alpha parameter, indicating higher value updating/learning rate, was associated with smaller distances between successive touches (GLMM, main effect of alpha; *p* = 0.046; *β*_alpha_ = −2.010; see Eq. 10 in Table 1), suggesting that animals that were more sensitive to outcomes in their choice behavior also showed less variability in their actions trial to trial. Additionally, this equation identified the sex difference in touch variability shown in Figure 1 (GLMM, main effect of sex; *p* = 0.018; *β*_sex_ = 2.512; see Eq. 10 in Table 1).

### Separate bouts of exploit choices are more overlapping than separate bouts of explore choices and more overlapping in females than males

We find that animals become less variable in their touch responses as a result of exploit states, following rewards, and in females in general compared with males. Why are animals generating less variable touches, decreasing the variability of their responses when there is no overt cue to target or reward benefit for doing so? One possibility supported by our analyses is that less variable responses reflect increased stereotypy induced during exploit states, reflecting reduced deliberative effort. Our results demonstrating that reward also decreases action variability suggests that reinforcement of a specific action pattern could contribute to the development of stereotypy during exploit states. If so, we might expect that separate “bouts” of exploit states would be more similar to each other, reflecting induced stereotypy that is released during transitions to exploration. In turn, separate bouts of explore behavior would be expected to be less similar to each other, potentially reflecting sampling of individual touch locations.

**Figure 3.**
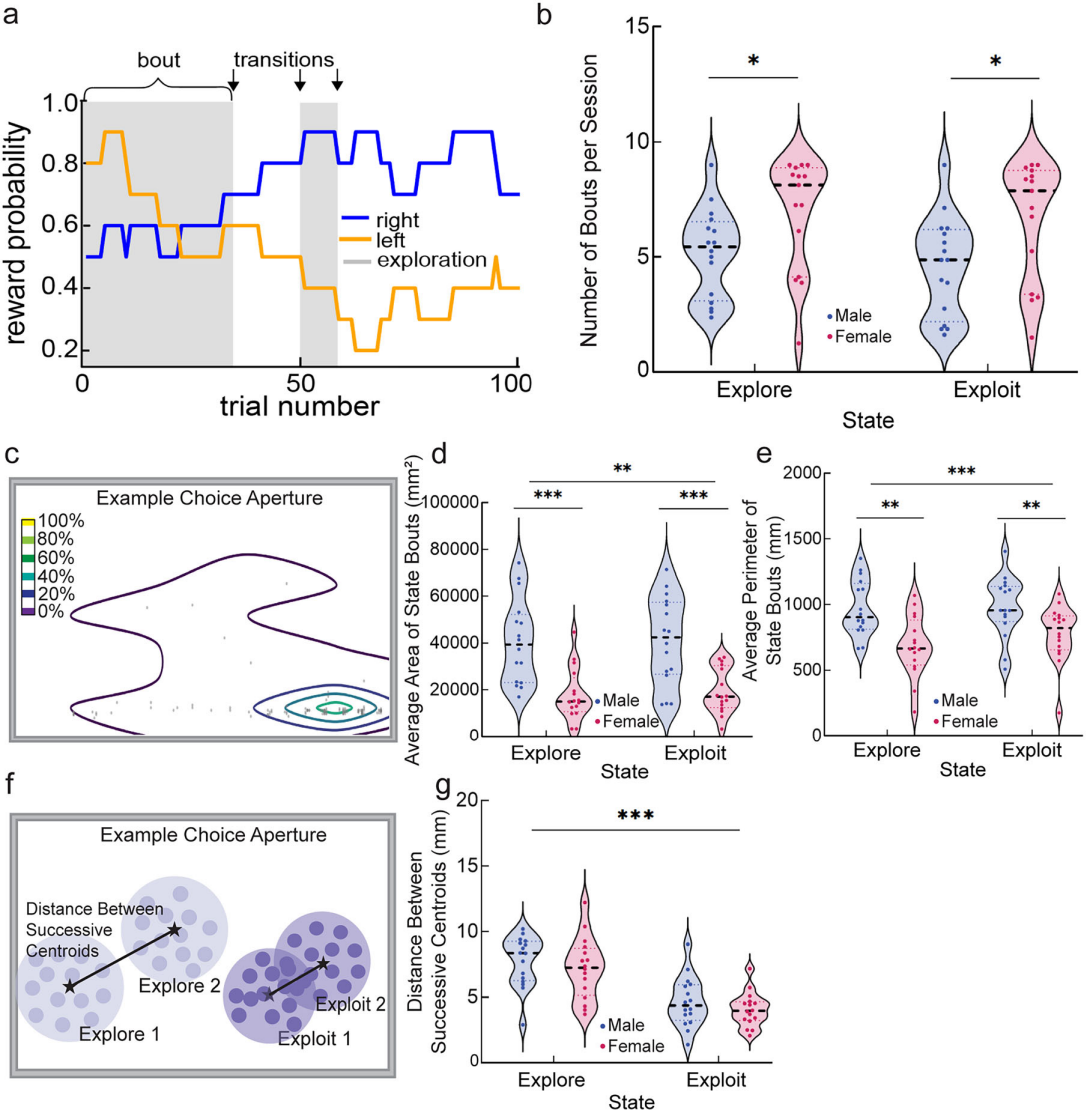
Bouts of exploit choices are closer to each other and occupy a smaller area than bouts of explore choices. ***a***, An example portion of a probability walk with explore trials shaded gray, illustrating state bouts and transitions between bouts. A “bout” is a period of touches within one HMM-defined behavioral state. Transitions between bouts are referenced with arrows as the state switches from explore to exploit or exploit to explore throughout. ***b***, The average number of bouts per session split by state and sex. No significant sex difference in the number of bouts per session. Red indicates female, and blue indicates male mice. In violin graphs, individual data points are data from one mouse averaged across all sessions. ***c***, An example 2D contour plot from Plotly Graphing Libraries fit over our nosepoke touch locations to visualize the density and range of choice responding. Small gray circles are nosepoke touches within the bout of response data. The color map corresponds with density of data points within each bin, where the darkest purple (outer bin) is the least dense contour bin, which is used to calculate the area and perimeter of the bout. ***d***, The average area of bouts split by state and sex. Exploit touches and females had significantly reduced area. Red indicates female, and blue indicates male mice. ***e***, Average perimeter of bouts split by state and sex. Exploit touches and females had significantly reduced perimeter. ***f***, Schematic depicting centroid shifts, where the Euclidean distance between two successive Mahalanobis centroids is calculated. Stars indicate example centroids associated with bouts, and black lines indicate the distance calculations between those centroids. ***g***, Centroid shifts split by state and sex. Centroid shifts were significantly smaller for exploit bouts. Red indicates female, and blue indicates male mice. *p < 0.05 and *p > 0.01; **p < 0.01 and **p ≥ 0.001; ***p < 0.001. Violin graphs depict median and quartiles of the dataset.

We separated each session into explore and exploit state “bouts” (Fig. 3*a*). A “bout” is defined as a period of touches within one state on a particular choice aperture. State transition trials from either explore to exploit or exploit to explore trigger a new “bout.” On average, mice complete 11.6 “bouts” per session due to switching between exploring (averaging 6.0 bouts) and exploiting (averaging 5.6 bouts; Fig. 3*b*). Females switch states more frequently than males (Fig. 3*b*, GLMM, main effect of sex in explore state; *p* = 0.038; *β*_state_ = −2.178; see Eq. 11 in Table 1; Fig. 3*b*, GLMM, main effect of sex in exploit state; *p* = 0.025; *β*_state_ = −2.367; see Eq. 12 in Table 1), complimenting the previous finding that duration of bouts differ across sex, where males explore for longer than females (Chen et al., 2021b). We then calculated the area and perimeter of touches associated with each state “bout.” “Bouts” of touches were plotted and overlaid onto 2D contour plots from Plotly Graphing Libraries (Fig. 3*c*). For each bout, OpenCV was used to capture the contours (bin traces) along continuous boundaries of the contour plots and calculate the area and perimeter for the outermost bin—which is recognized as the outer range of nosepoke responses.

Exploit bouts occupied a smaller area (mm^2^) on the screen and were less variable than explore bouts (Fig. 3*d*, GLMM, main effect of state; *p* = 0.008; *β*_state_ = −2.661; see Eq. 7 in Table 1). Female mice used significantly less area of the screen per bout than males (Fig. 3*d*, GLMM, main effect of sex; *p* < 0.001; *β*_sex_ = 4.731; see Eq. 7 in Table 1). The model used included a significant interaction term between state and sex (Fig. 3*d*, GLMM, interaction state/sex; *p* = 0.004; *β*_state*sex_ = −2.875; see Eq. 7 in Table 1). Regarding the perimeter of the touchscreen choice apertures used by the mice, exploit bouts occupied a smaller boundary (mm) on the screen and were less variable than explore bouts (Fig. 3*e*, GLMM, main effect of state; *p* < 0.001; *β*_state_ = −7.161; see Eq. 8 in Table 1). Female mice occupied a smaller boundary on the screen and were less variable than bouts by male mice (Fig. 3*e*, GLMM, main effect of sex; *p* < 0.001; *β*_sex_ = 3.643; see Eq. 8 in Table 1). The model used included an interaction term between state and sex, which was not significant (Fig. 3*e*, GLMM, interaction state/sex; *p* = 0.125; *β*_state*sex_ = −1.535; see Eq. 8 in Table 1), supporting independent mechanisms for decreased action variability by exploit states and female sex in mice.

Each new bout of responding includes its own centroid, and these centroids may minutely move across the screen throughout a session, allowing us to compare the similarity of separate exploit bouts and separate explore bouts to each other. Figure 3*f* shows how the distance between separate bouts of each state is calculated using the *x* and *y* centroid coordinates—as determined by the Mahalanobis analysis. Distances between centroids for successive exploit bouts were smaller and less variable than distances between centroids for successive explore bouts (Fig. 3*g*, GLMM, main effect of state; *p* < 0.001; *β*_state_ = 8.964; see Eq. 6 in Table 1). This did not differ by sex (Fig. 3*g*, GLMM, no main effect of sex; *p* = 0.991; *β*_sex_ = −0.011; see Eq. 6 in Table 1). The model used included an interaction term between state and sex, which was significant (Fig. 3*g*, GLMM, state/sex; *p* = 0.038; *β*_sex*state_ = 2.078; see Eq. 6 in Table 1). We found that touches occurring during one bout of exploration were farther and more variable in distance from other bouts of exploration compared with more similar touch patterns across bouts of exploitation. Given that mice are using more overall screen space during explore than exploit trials, this further increases the likelihood that during exploration, mice may be sampling individual touch locations over and above sampling just the left/right options we define. In contrast, exploit states reflect a return to a stereotyped selection of a similar area of the screen.

## Discussion

The explore/exploit trade-off is a fundamental property of choice selection during reward-guided decision making. Explore and exploit states are mediated by distinct neural circuit activity and reflect slower versus faster decision processes (Ebitz et al., 2018, 2019, 2020; Tervo et al., 2021; Bolkan et al., 2022; Wang et al., 2023; Wyatt et al., 2023). These neural findings suggest that explore/exploit balance may also be reflected, and measurable, in the execution of a task. Using touchscreen operant chambers in mice, we asked whether explore/exploit balance governed the variability of actions during decision making, finding independent effects of (1) explore/exploit state, (2) prior reward, and (3) sex on increasing similarity of touches. These data suggest that multiple independent mechanisms regulate the variability of actions associated with choices and that the explore/exploit state is visible at the level of motor performance.

Exploration and deliberation processes involve the subject surveying options (Payne et al., 1993; Gilbert and Wilson, 2007; Rangel et al., 2008). Deliberation is physically expressed through pausing, slower decision making, and “vicarious trial-and-error” behavior, reflecting forward thinking and prospective deliberation (Tolman, 1939, 1948; Johnson and Redish, 2007; Dolan and Dayan, 2013; Redish, 2016; George et al., 2023). We observed that explore touches happen significantly closer to the center of the screen than exploit touches, which implies animals are approaching exploratory choices between the two apertures, rather than from off to one side. Though there is no video tracking to supplement this specific experiment, we suggest future experiments implement video tracking and analysis to further explore the kinetics of a choice including approach trajectory to the touchscreen and vicarious trial-and-error behaviors prior to nosepoke. In addition, we found that touches occurring during one “bout” of exploration were farther from other bouts of exploration compared with exploit bouts. Given that mice are using more overall screen space during explore than exploit trials, this suggests mice may be exploring individual touch locations across the screen over and above sampling just the left/right options we define. Self-directed exploration may reflect an increasingly fine-grained goal–directed search for the most rewarding action.

A potential confound between explore/exploit state and action variability is that exploit actions are more likely to be reinforced. However, exploit states and prior reward independently reduced action variability. This suggests that while reward may cause trial-to-trial adjustments in responding on the touchscreen, reward does not overpower the state effect. Reward-triggered changes in response variability may be a function of individual reward sensitivity. Animals with a higher learning rate derived from a reinforcement learning model showed smaller distances between successive touches, suggesting that reward sensitivity varying across individuals is associated with increased action precision. This effect was larger in females than in males, highlighting sex as a third independent factor governing choice precision.

Though the primary focus of this paper was to investigate the kinetics of choice response across explore/exploit state and sex, another promising avenue of research is the impact of decision difficulty on motor responding and variation in both explore and exploit states. In humans, reduced reaction time is often seen with a decrease in task difficulty in both reward-guided and perceptual decision making tasks (Churchland et al., 2008; Siedlecka et al., 2021; Suarez et al., 2021) and with increased stability of environmental conditions (Parrington et al., 2015). Across species, perceptual decision making tasks reveal that higher certainty, less difficult decisions are more motorically precise, even when the decision does not require motor accuracy (Wolpert and Landy, 2012; Palser et al., 2018; Follman et al., 2023; Sanchez et al., 2024). Exploit choices happen faster in comparison with explore choices (Ebitz et al., 2018; Chen et al., 2021b, 2023), and stereotyped performance of a behavior has previously been linked to a lack of deliberation (Mitchell and Etches, 1977; Foster, 1998; Graybiel, 2008; Smith and Graybiel, 2016). Our findings are broadly consistent with the idea that exploit choices reflect behavioral automation with repetitive action performance, while explore choices reflect deliberation with more variability in the timing and performance of choices.

The data in this manuscript were previously used to reveal a sex difference in the balance of explore/exploit strategies (Chen et al., 2021b). Because male and female mice employ different strategies in the two-arm spatial restless bandit task, we sought to test whether motor responses associated with the different strategies were physically different in distribution and spatial location. We found that actions were more precise in females compared with males, independent of the impact of explore/exploit state and reward experience, suggesting individual differences regulating action variability over and above moment to moment features of the task. However, not all explore/exploit differences were sex different. In particular, there was no sex difference in how close animal responses were to the center of the screen during exploration. This suggests that the overall deliberative process of an exploratory decision is probably similar across sexes, but the sequential execution of these decisions is more similar in females than males. Overall these findings agree with a growing literature that finds male decision and/or motor behavior to be more variable than females in rodents (Chen et al., 2021a; Levy et al., 2023) and humans (Dosenbach et al., 2017). This may be due to chromosomal and/or hormonal influences on action selection circuits, including the striatum (Becker and Chartoff, 2019; Grissom and Reyes, 2019; Grissom et al., 2024), but further work is needed.

Touchscreens are increasingly used not only by rodent researchers but by people working with humans via smartphone-mediated ecological assessments or other touchscreen-enabled devices used in clinics such as touchscreen tablets. Our analysis reveals a powerful way to evaluate the distribution and consistency of motor behaviors in choice responding when using touchscreens. Motor abnormalities are a common feature across patients with psychosis (Walther and Mittal, 2017), autism (Mosconi and Sweeney, 2015; Mody et al., 2017), and depression (Sobin and Sackeim, 1997), and explore/exploit trade-offs reveal neuropsychiatric influences (Addicott et al., 2017; Wyatt et al., 2023). Motor abnormalities are also central to neurodegenerative conditions such as Parkinson's disease, which has also been linked with cognitive differences in reward processing (Künig et al., 2000; Schott et al., 2007; Rowe et al., 2008; Gleichgerrcht et al., 2010; Kapogiannis et al., 2011; O’Callaghan et al., 2014; Perry and Kramer, 2015; du Plessis et al., 2018), raising the possibility of a link between these features measurable via touchscreens. The increasing prevalence of touchscreen technology testing in human neuropsychiatric research raises the distinct possibility of analyses of touch responses (Azenkot and Zhai, 2012; Miller, 2012; Gosling and Mason, 2015; Harari et al., 2016; Intarasirisawat et al., 2019) as a novel cross-species translational measure of explore/exploit trade-offs, as well as identifying developing stereotypy and deviations from baseline motor learning and control data.

## Synthesis

Reviewing Editor: Mark Laubach, American University Decisions are customarily a result of the Reviewing Editor and the peer reviewers coming together and discussing their recommendations until a consensus is reached. When revisions are invited, a fact-based synthesis statement explaining their decision and outlining what is needed to prepare a revision will be listed below. The following reviewer(s) agreed to reveal their identity: NONE.

Thank you sending your work to eNeuro. Your paper was reviewed by two experts and their reviews are given below in full. Please revise your paper to address all points that were raised.

Please consider submitting a visual abstract with your revised manuscript. This helps with promoting your work by social media.

Please also consider providing analysis code and example data either as Extended Data or through a public repository.

Reviewer 1

The authors provide a comprehensive set of analyses exploring how precision in the touchscreen operant chamber environment map on to exploring/exploiting behavior during a spatial 2-arm bandit task. They extend their previously published work (Chen et al, 2021) exploring how choice responses in the bandit task map on to exploring/exploiting behavior. To measure spatial responding, the authors provide a variety of methodologies including distance from the center, Euclidian distance spacing, Mahalanobis distance. They also explore the total area of response locations by plotting contour plots. The authors used these methodologies to explore the transition states between uncertain exploring behavior to more certain exploiting behavior. The authors further explore the differences between male and female mice to understand sex differences in transitioning between states and screen responding.

I am huge fan of the development of new behavioural methods and analyses, which often takes a back seat to development of techniques for neural manipulation and monitoring, which are limited without good behavior. Overall, the manuscript is a valuable resource that provides meaningful statistical methodologies for better understanding rodent behaviour in the touchscreen operant chamber. I appreciated the well detailed methodology and explanation of all the metrics. The authors approach to including multiple types of spatial analyses provides further justification of their claims.

My main issue is with the presentation of the study in the manuscript. The Introduction, for example, is quite jargon-y and dense, so will be a hard read for the non-expert. This is a shame, because it limits the reach of the study. I think with some re-writing the manuscript would become more accessible to a wider audience, outside of this specific subfield. Furthermore, some of the experimental choices (e.g. choice of the touchscreen bandit task) lacked sufficient explanation and justification. Finally, the potential impacts of the new touch analysis could be better expanded upon.

Introduction:

-The Introduction needs a clear statement of the main question of the study, why it is important for us to know the answer, and the hypothesis.

-The authors do not present sufficient information describing the distinction between exploring and exploiting state and how it relates to other cognitive constructs such as learning and decision confidence/certainty. Does explore just mean the animal is at an early stage of learning, and exploit mean well-learned behaviour? There is no definition of these central constructs, except to refer the reader to a description and diagram of the hidden Markov model (Figure 1c). But Figure 1c provides little information. Is the explore and exploit in those traces? The reader needs a lot more help understanding this.

-It is unclear in the current draft how certainty/uncertainty contribute to motor precision. The authors should add a sentence or two to provide further context.

-The authors suggest that uncertainty can be controlled parametrically in perceptual tasks. Can this be elaborated on? Was it not of interest in this study to take advantage of this fact and manipulate parameters to affect certainty?

-The way this is written suggests that reward-guided decision making and perceptual decision making are separate. But these are nested processes. Perhaps this is just another way of saying early versus late learning (early perceptual learning is followed by overlapping stimulus-reward mapping processes)? Is it surprising that when animals are learning to discriminate they center themselves to view and compare both stimuli, and therefore respond in the center? And when they've done the perceptual learning and now just need to respond to the rewarded stimulus, they can approach the stimuli from any angle and therefore their responses are less central? In a paper about understanding the factor influencing behavioral performance, it seems of primary importance to discuss such relationships.

-Are there other ways to look at the kinetics of choice in a touchscreen environment in addition to screen touches? For example do the authors have video recordings they could analyze? That seems like it might be a simple way to get some information about, e.g., exploit v explore, by analyzing, e.g., vicarious trial-and-error behavior. It could also be used to validate the hidden Markov model.

-The authors reference their previously published research as a rationale for evaluating sex differences. There needs to be a clear rationale for why this was looked at in this manuscript. In its current form, this is not well justified. The authors say there are differences in strategy in the two-arm restless bandit task. What are the differences?

-The authors do not provide any details in the introduction as to the behavioural task they will be using. Particularly important to explain is how the task is suited to answer the question of the study. Is the answer in the shifting reward contingencies? It's not clear how these are used in the present study.

Methods:

-The authors acknowledge the data in this manuscript are also used in Chen et al (2021b). I noticed that the methodology is comparable to Chen et al (2021a), with the exception of the task design, which used different stimuli. Were the mice also used in Chen et al (2021a) to develop the paradigm?

-The authors do not provide sufficient information describing the touchscreen two-arm bandit task. The authors should provide a full outline of the method, rather than referring to Chen et al (2021b). The paper should be interpretable without the need to review other manuscripts.

-I found the insight that mice tended to prefer central screen responses interesting. This preference served as the basis for the distance from center metric reported in the manuscript. Has this preference been observed in other studies? Additional exploration of the cause of this would provide additional justification for the use of the method. I provided some thoughts above that may or not be useful in this regard.

-The authors mention that some of the data in the Euclidian analysis was lost due to transitions between screen locations. As a percentage, approximately how much was lost to these transitions?

-I appreciate the authors' attention to detail including the information regarding which packages and CV functions were used. Given this level of detail, it was surprising that the authors did not provide a link to a GitHub repository containing the scripts that were used for the analysis. Given the highly computation/statistical nature of the paper, access to scripts through a repository would be a significant improvement and contribution.

-Were additional data collected or examined in this experiment, including response latency? Seems response times would be quite useful in these analyses.

Results:

-Why was this study done with just white squares and not images as in Chen et al 2021a? In the current manuscript, the authors present a task variant with white squares as the stimuli. I wonder if "precision" is the right word for responding within a large white square on the screen when no particular location within that square is deemed the target or trained as correct. Instead precision seems to be defined as the consistency with which the animal repeats its particular response. Would you expect a different result in the task from Chen et al 2021a were used?

-Is the precision in response observed in exploiting behavior due to motor stereotypy. Does this account for any variance outside of decision confidence?

-How many bouts did animals have on average in a session. How much switching between exploring/exploiting was there?

Discussion:

-The authors focus primarily on touch input as the main metric of precision. Is touch the only relevant form of precision in the operant chamber. How does touch relate to measures such as reaction time? Is there a relationship?

-The authors suggest that exploring behaviour involves pausing and trial and error behaviour. Wouldn't latency data further support be modelling these states?

-Could the authors include references to literature in humans regarding motor responding and motor variation due to decision difficulty?

-The final paragraph suggesting the approach be used in human smartphone research could be improved. First, why only smartphone and not any touchscreen-enabled device (ipads, for example, which are also used widely in clinics)? The authors suggest motor abnormalities are common in patients with a variety of conditions. These motor abnormalities are different from decision confidence, so can the authors say more about what these motor 'precision' measurements might look like in patients. Also there is a focus on neuropsychiatric conditions but what about neurodegenerative conditions like Parkinsons?

More minor:

The authors have copied the same sentence in the introduction and discussion. The following sentence is repeated exactly as written:

Perceptual decision-making tasks reveal that higher certainty decisions are more motorically precise, even when the decision does not require motor accuracy (Follman et al., 2023; Palser et al., 2018; Sanchez et al., 2024; Wolpert and Landy, 2012).

Chen et al 2021b and Chen et al 2021c are duplicate references.

Chen et al 2023a and Chen et al 2023b are duplicate references.

Page 10 Figure 1j does not exist

The word "data" is plural, so e.g., "these data" not "this data", throughout.

Page 4 "eLife publication" needs to be fixed.

Page 12: Autoshaping is a pavlovian process. It's only called autoshaping because Brown and Jenkins wanted to shape pecking behavior and found if they set up a pavlovian relationship between the stimulus and reward the pigeon would approach and peck the stimulus, seemingly auto-shaping the instrumental behavior they were seeking to train.

Reviewer 2

Authors present a new analysis of previously-published data and supporting analytical methods to measure the degree to which touchscreen touch precision and timing of touches (in mice) reflects explore/exploit states, prior reward, and other individual factors such as sex. Authors present interesting results employing a variety of approaches: HMM categories of explore/exploit, their RLCK reinforcement learning model, Euclidean and Mahalanobis distance analyses, distance from center of screen, and distance between bouts of successive touches, to name the main ones. Authors found that actions are more precise in exploit states (far left and far right) than explore states (more central touches). This was independent of the effect of previous reward on touch location. Also independently, touches were more precise in females compared to males. Overall, I was convinced by the rigor of the analytic methods and the usefulness of its contribution to the scientific community (i.e., it is exciting to take advantage of the location of screen touch information across sex). I offer suggestions to improve communication of its conceptual advance and perhaps to provide a clearer interpretation.

I found mention of perceptual tasks, decision confidence, and choice certainty in the Introduction tangential and distracting. Though it is true that perceptual choices involving greater certainty are motorically more precise, the authors present a very different (bandit) task and analysis here that may or may not extend to such cases. In addition, in those perceptual tasks authors typically employ a behavioral readout of such certainty (i.e., post-choice temporal wagering for reward) that can result in a more objective measure of statistical confidence. After reading the Introduction I am left anticipating that authors will similarly provide us with the same kind of confidence report in the present experiment. It may be best to leave this out of the Introduction and stick to descriptions of the explore-exploit tradeoff.

I understand that these are new analyses of previously published data. Indeed, authors refer to the computational models fit to the mouse data including the validated hidden Markov model (HMM) and the RLCK reinforcement learning model (acronym not provided here in this paper). Though there is an emphasis on such parameter-level analysis, I was disappointed there was not more that could be done with primary behavioral data. For example, do authors have latency data in these mice (to choose, to collect reward, etc.) that could augment/confirm the touch analyses? One would predict that the exploit state would be associated with quicker reward retrieval, but we do not have this information.

Authors state that a stepwise GLM was used (GLM was also not spelled out in first instance). Overall, I found the GLM analyses inadequately specified. I am left to imagine the formulas the authors used as there are no reports of Beta coefficients, and no description of how they coded the variables. I suggest adding a section describing this in a Data Analysis section including the function they used to run the models along with tables to support their analysis with all the appropriate values / GLM results. Relatedly, the bars and asterisks above violin plots suggest that authors ran a NHST (t-test or ANOVA) for these comparisons when in fact these were all GLM results with significant Beta coefficients. Descriptions should be changed to more clearly convey that authors ran GLM models with regressors, (i.e., potential predictors).

Minor:

Two of the Chen et al. papers appear twice in the references list. A Gonzalez et al. paper appears in references list but is not cited in the main text.
